# Coupling of Alzheimer’s Disease Genetic Risk Factors with Viral Susceptibility and Inflammation

**DOI:** 10.14336/AD.2023.1017

**Published:** 2024-10-01

**Authors:** Can Cao, Gaoshuang Fu, Ruodan Xu, Ning Li

**Affiliations:** Department of Biomedical Engineering and Technology, Institute of Basic Theory for Chinese Medicine, China Academy of Chinese Medical Sciences, Beijing, 100700, China.

**Keywords:** Alzheimer’s disease;, risk genes, viral susceptibility, inflammation

## Abstract

Alzheimer’s disease (AD) is a neurodegenerative disease characterized by persistent cognitive decline. Amyloid plaque deposition and neurofibrillary tangles are the main pathological features of AD brain, though mechanisms leading to the formation of lesions remain to be understood. Genetic efforts through genome-wide association studies (GWAS) have identified dozens of risk genes influencing the pathogenesis and progression of AD, some of which have been revealed in close association with increased viral susceptibilities and abnormal inflammatory responses in AD patients. In the present study, we try to present a list of AD candidate genes that have been shown to affect viral infection and inflammatory responses. Understanding of how AD susceptibility genes interact with the viral life cycle and potential inflammatory pathways would provide possible therapeutic targets for both AD and infectious diseases.

Alzheimer’s disease (AD), a neurodegenerative disorder that causes progressive cognitive decline and memory loss, presents formidable challenges for individuals, families, and healthcare systems worldwide. According to estimates by World Health Organization (WHO), there are currently 55 million people living with dementia, and this number is predicted to reach 139 million by 2025 [1]. AD accounts for approximately two-thirds of all dementia cases, with its prevalence increasing exponentially with age, particularly among individuals aged 65 and older [1]. At a pathological level, AD is characterized by the accumulation of beta-amyloid (Aβ) plaques and tau tangles, which are two prominent hallmarks of AD pathology. These pathological entities disrupt neural communication and impair synaptic function, ultimately leading to neuronal degeneration and death. As individuals age, their susceptibility to the buildup of Aβ plaques and tau tangles in the brain increases [2]. A growing body of evidence indicates that inflammation, including the inflammation following viral infection, contributes to AD progression. Inflammatory mediators can alter synaptic function and drive neurons and glial cells to produce more Aβ, further affecting cognitive performance in AD patients [3].

It is widely acknowledged and supported by research that AD has a high heritability, estimated to be approximately 60-80% [4]. With the application and expansion of large-scale genome-wide association studies (GWAS), several AD susceptibility genes have been identified [5-7], providing more clues to explain the underlying pathophysiological process of AD and to propose novel therapeutic targets for this debilitating disease. In addition, the viral hypothesis of AD has gained prominence, suggesting that viral infection may serve as a potential trigger of neurodegenerative diseases [8]. While the precise mechanisms by which viral particles influence the onset and progression of AD remain unclear, research has demonstrated the neurotoxic properties of viral components that could potentially drive AD pathological processes. For instance, human immunodeficiency virus (HIV) may accelerate AD-like pathology in HIV-infected patients who develop neurological disorders associated with Aβ plaque deposition and tau protein aggregation [9]. In addition, the potential impact of severe acute respiratory syndrome coronavirus 2 (SARS-CoV-2) on AD has recently garnered significant attention. Patients with AD are a vulnerable group susceptible to viral diseases such as coronavirus disease 2019 (COVID-19) [10, 11], and some findings suggest that individuals with a history of COVID-19 may experience an acceleration of AD-related symptoms and pathology, both in the short term and long after recovering from the infection [12]. One common explanation for the aforementioned is that viral infection may induce an abnormal immune response that persists for years and eventually produces neurological damage [13].

Although how an AD susceptibility gene leads to increased vulnerability to viral infection remains largely unexplored, incremental clinical evidence has shed light on the role of viral infection-induced inflammation in accelerating AD progression. The current review aims to correlate AD risk genes with viral infection and the associated inflammatory responses to enable a better understanding of the pathogenesis and progression of AD with implications of therapeutic targets.

**Figure 1.**
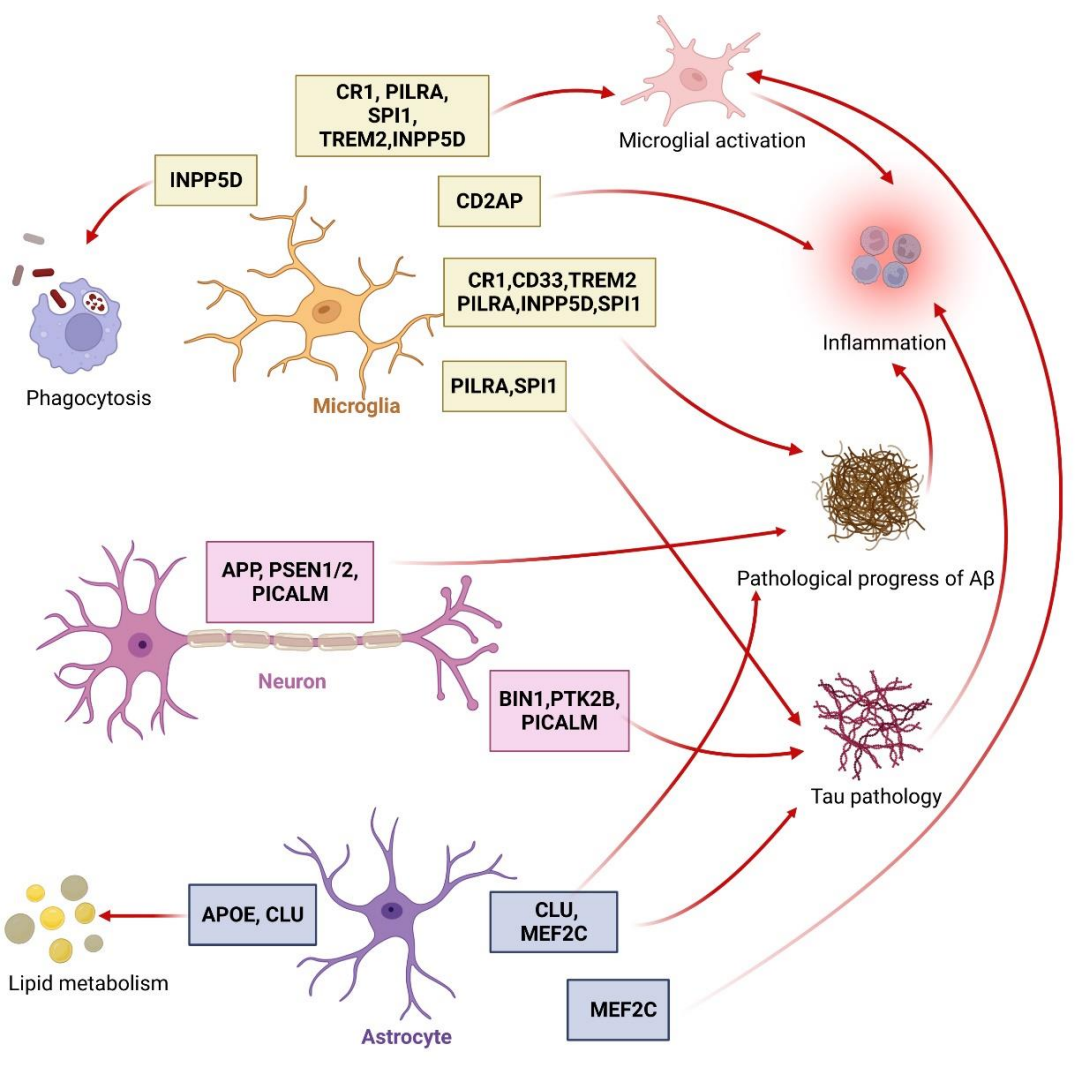
Schematic illustrations of genes linked to neuropathology in AD. Genes associated with microglia are presented in yellow boxes, genes associated with neurons are listed in pink boxes, and those associated with astrocytes are shown in purple boxes. Red arrows represent facilitated actions. AD, Alzheimer’s disease; Aβ, β-amyloid; APOE, apolipoprotein E; APP, amyloid precursor protein; BIN1, bridging integrator 1; CD2AP, the CD2-associated protein; CLU, clusterin; CR1, complement receptor type 1; INPP5D, inositol polyphosphate-5-phosphatase; MEF2C, myocyte enhancer factor 2C; PICALM, phosphatidyl inositol- binding clathrin assembly protein; PILRA, paired immunoglobulin-like type 2 receptor A; PTK2B, Protein tyrosine kinase 2 beta; PSEN1/2, presenilin1/presenilin2; SHIP1, src homology 2 domain-containing inositol phosphatase 1; SPI1, salmonella pathogenicity island 1; TREM2, triggering receptor expressed on myeloid cells 2.

**Figure 2.**
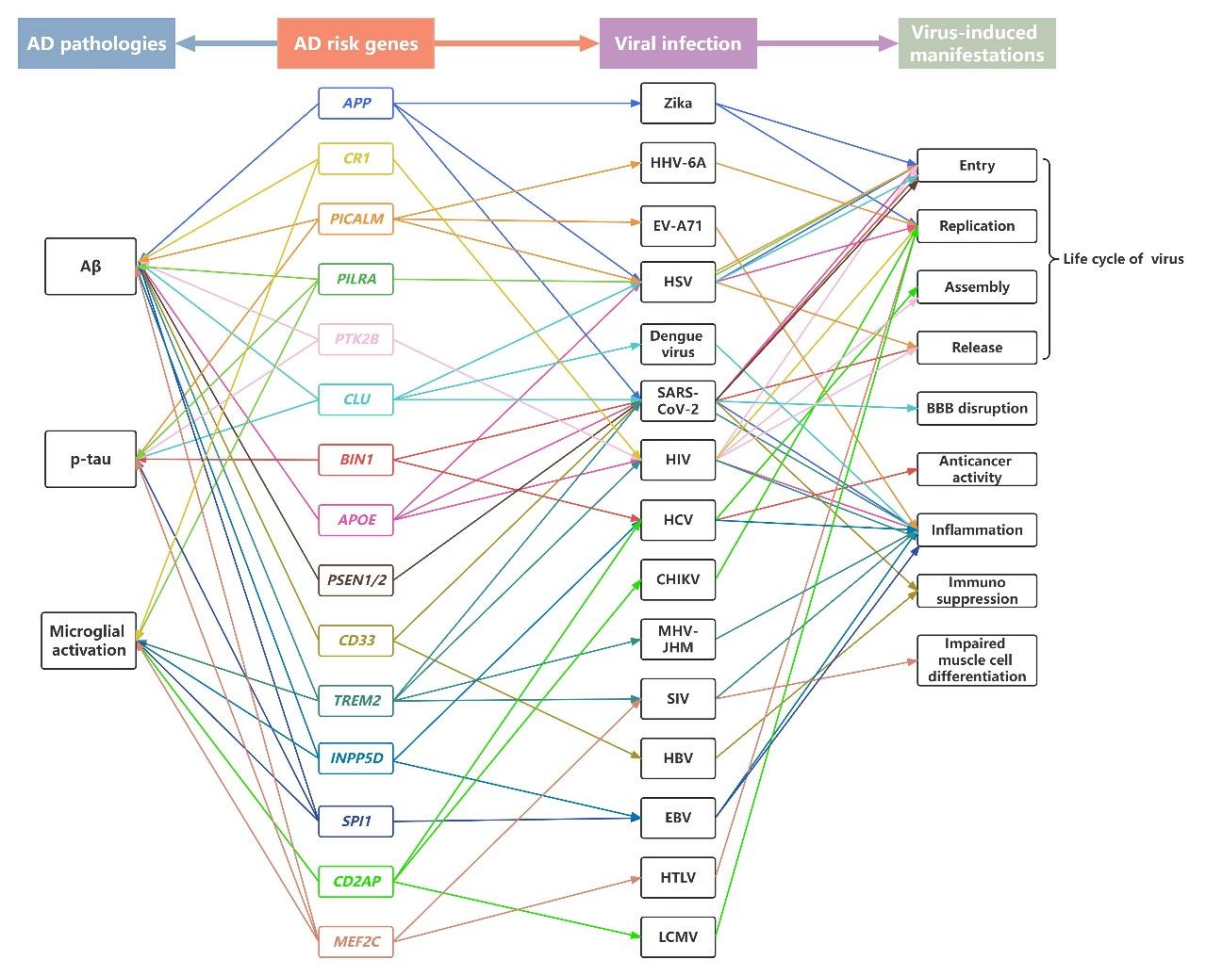
Effects of AD risk genes on AD pathology, viral infections and the associated manifestations. Colored boxes represent different AD risk genes, and colored arrows refer to the connection of each gene with AD pathologies and viruses marked with the corresponding color. AD, Alzheimer’s disease; Aβ, β-amyloid; *APOE*, apolipoprotein E; *APP*, amyloid precursor protein; *BIN1*, bridging integrator 1; *CD2AP*, CD2-associated protein; *CD33*, cluster of differentiation 33; CHIKV, chikungunya virus; *CLU*, clusterin; *CR1*, complement receptor type 1; EBV, Epstein-Barr virus; EV-A71, enterovirus A71; HBV, hepatitis B virus; HCV, hepatitis C virus; HHV-6A, human herpesvirus 6A; HIV, human immunodeficiency virus; HSV, herpes simplex virus; HTLV, human T-cell leukemia virus type; *INPP5D*, inositol polyphosphate-5-phosphatase; LCMV, lymphocytic choriomeningitis virus; *MEF2C*, myocyte enhancer factor 2C; MHV-JHM, JHM strain of mouse hepatitis virus; *PICALM*, phosphatidyl inositol- binding clathrin assembly protein; *PILRA*, paired immunoglobulin-like type 2 receptor A; PSEN1/2, presenilin1/presenilin2; PTK2B, Protein tyrosine kinase 2 beta; SARS-CoV-2, severe acute respiratory syndrome coronavirus 2; SIV, simian immunodeficiency virus; *SPI1*, salmonella pathogenicity island 1; *TREM2*, triggering receptor expressed on myeloid cells 2.

## 1. Genetic risk factors for AD associated with viral infection

**Figure 3.**
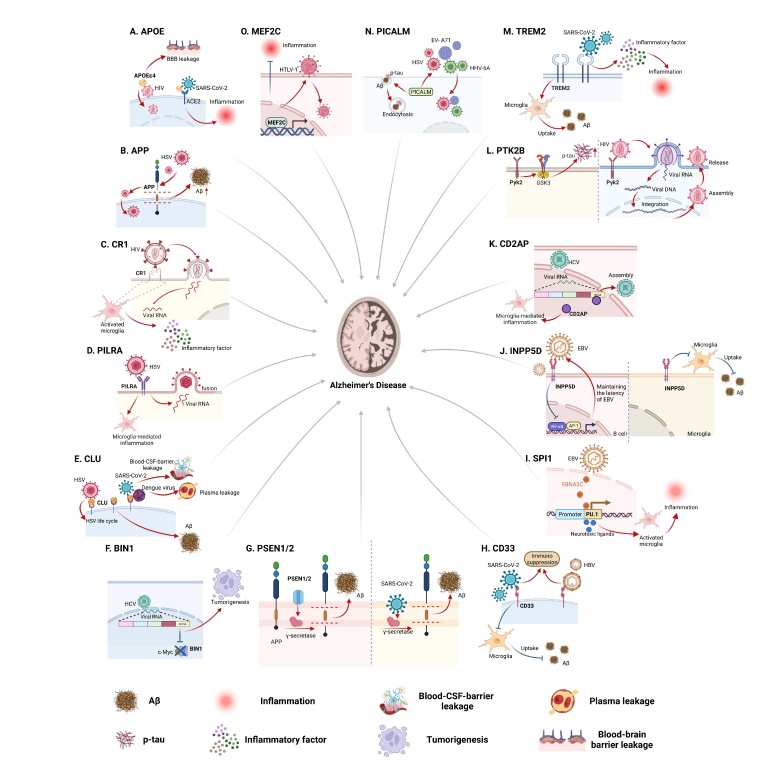
Effects of gene-virus interactions that accelerate viral infection and AD progression. Red arrows represent facilitated actions, and blue "T" lines represent inhibited actions. (A) APOE, APOE4 promotes the invasion of SARS-CoV-2 and HIV to activate the inflammatory response and damage the BBB. (B) APP, APP accelerates HSV invasion and HSV interferes with APP processing to increase Aβ. (C) CR1, CR1 enhances HIV replication and activates inflammation. (D) PILRA, PILRA assists HSV entry into the cell and drives neuroinflammation. (E) CLU, CLU is involved in the HSV life cycle, accelerating the invasion of SARS-CoV-2 and dengue viruses, damaging the blood-CSF-barrier, leading to plasma leakage. (F) BIN1, HCV NS5A disrupts the binding of BIN1 and c-Myc, thereby interfering with the tumor suppressive effect of BIN1. (G) PSEN1/2, SARS-CoV-2 enhances γ-secretase activity through PSEN1/2 to promote the cleavage of APP to Aβ. (H) CD33, Immunosuppressive effects of CD33 enhance SARS-CoV-2 and HBV infection and impede Aβ uptake by microglia. (I) SPI1, PU.1, activates microglia-mediated inflammation in response to neurotoxic ligand stimulation; it binds to EBNA3C of EBV to enhance transcription. (J) INPP5D, INPP5D inhibits Aβ uptake and participates in the inhibition of EBV-induced NF-kB response. (K) CD2AP, CD2AP promotes microglia-mediated CNS inflammation and binds with NSP5A of HCV to promote viral assembly. (L) PTK2B, Pyk2 facilitates the generation of p-tau and assists HIV entry, assembly, and release. (M) TREM2, TREM2 promotes Aβ uptake and interacts with SARS-CoV-2 to initiate inflammatory responses. (N) PICALM, PICALM facilitates the internalization of Aβ and p-tau and contributes to the invasion of EV-A71, HSV, and HHV-6A. (O) MEF2C, MEF2C inhibits inflammation and promotes HTLV-1 infection. Aβ, β-amyloid; ACE2, angiotensin-converting enzyme 2; APOE, apolipoprotein E; AP-1, activator protein 1; APP, amyloid precursor protein; BBB, blood-brain barrier; BIN1, bridging integrator 1; CD2AP, CD2-associated protein; CD33, cluster of differentiation 33; CHIKV, chikungunya virus; CLU, clusterin; CR1, complement receptor type 1; CSF, cerebrospinal fluid; EBV, Epstein-Barr virus; EBNA3C, EBV nuclear antigen 3C; EV-A71, enterovirus A71; GSK3, glycogen synthase kinase3; HBV, hepatitis B virus; HCV, hepatitis C virus; HHV-6A, human herpesvirus 6A; HIV, human immunodeficiency virus; HSV, herpes simplex virus; HTLV-1, human T-cell leukemia virus type 1; INPP5D, inositol polyphosphate-5-phosphatase; MEF2C, myocyte enhancer factor 2C; NF-κB, nuclear factor kappa-light-chain-enhancer of activated B-cells; PICALM, phosphatidyl inositol- binding clathrin assembly protein; PILRA, paired immunoglobulin-like type 2 receptor A; PSEN1/2, presenilin1/presenilin2; p-tau, phosphorylated tau; Pyk2, proline-rich tyrosine kinase 2; SARS-CoV-2, severe acute respiratory syndrome coronavirus 2; SHIP1, src homology 2 domain-containing inositol phosphatase 1; SPI1, salmonella pathogenicity island 1; TREM2, triggering receptor expressed on myeloid cells 2.

By using GWAS, numerous genetic variants associated with AD have been identified (Fig. 1), some of which have also been shown to directly or indirectly influence the life cycle of various viruses (Fig. 2 and Table 1). Meanwhile, along with the observation that AD patients are more susceptible to certain viruses, viral infections have been demonstrated to promote the process of Aβ deposition, tau phosphorylation and inflammation, contributing to the advancement of AD. The relationship between AD risk genes and the vulnerability of AD to viral infections is currently becoming evident (Fig. 3); however, the molecular interpretation remains to be revealed.

### 1.1 Apolipoprotein E (APOE)

#### 1.1.1 Molecular information and biological function of APOE

*APOE* is a gene that encodes a protein involved in lipid metabolism in the brain. There are three common *APOE* subtypes: ε2, ε3, and ε4. The ε4 subtype is associated with an increased risk of developing AD and the relationship between this risk and the number of ε4 alleles is dose dependent. In contrast, the ε2 subtype is protective against AD, with lower Aβ accumulation and a reduced risk of cognitive decline in the elderly. However, some estimates suggest that individuals with one copy of the *APOE* ε4 allele have a 2- to 3-fold increased risk of developing AD, while those with two copies have a 12- to 15-fold increased risk compared to individuals without the *APOE* ε4 allele [14]. The physiological function of APOE is to regulate the metabolism of lipids such as cholesterol and triglycerides. In the brain, APOE is synthesized mainly by astrocytes and secreted into the extracellular space, where it associates with lipids to form lipoprotein particles [15]. Several studies have suggested that *APOE* ε4 carriers have increased deposition of Aβ, one of the AD pathology hallmarks. *APOE* ε4 has also been associated with reduced glucose metabolism and decreased brain volume in certain regions of the brain affected by AD, such as the hippocampus [16]. In association with inflammation, a study found that *APOE* ε4 was able to activate calcium-dependent cytosolic phospholipase A2 (cPLA2) in astrocytes, leading to enhanced cPLA2 release and downstream neuroinflammatory cascades [17]. In addition, oxidative stress markers were increased in *APOE* ε4-expressing cells, suggesting that *APOE* ε4-induced cPLA2 activation also potentiated oxidative stress [17]. Hence, APOE ε4 appears to trigger neuroinflammatory and oxidative stress responses *via* cPLA2 activation.

**Table 1:** Genetic risk factors of AD and their associated viral infection and inflammation.

Gene	Functions	Putative mechanisms	Viral infection	Inflammatory responses	References
*APOE*	Regulation of lipid metabolism	Impaired BBB function, increased deposition of Aβ and reduced glucose metabolism	HSV; HIV; SARS-CoV-2	Activating neuroinflammation via calcium-dependent cPLA2	[17, 19, 20, 168]
*APP*	Aβ production	Enhancing Aβ production and deposition	Zika; HSV; SARS-CoV-2	Activating microglial and astrocytic cells	[27, 29, 31, 33]
*CR1*	Ligand binding and complement regulation	Deposition of Aβ and neuroinflammation	HIV	Activating microglial cells	[38-41]
*PILRA*	Regulation of microglial activation, phagocytosis, and synaptic pruning	Associated with the enrichment of Aβ	HSV	Activating microglial cells	[43, 44, 48, 49]
*CLU*	Regulation of lipid metabolism, apoptotic, inflammation and Aβ	Accelerate the spreading of tau aggregates and participating in deposition of Aβ	HSV; Dengue virus; SARS-CoV-2	Participating in viral life cycle and mediating neurotropism	[53-55, 58, 59]
*BIN1*	Mediating endocytosis and membrane remodeling	Promoting tau aggregation and phosphorylation	HCV; SARS-CoV-2	Regulating tau pathology triggers neuroinflammation	[62, 65-67, 69]
*PSEN1/2*	Regulating synaptic functions, cell survival pathways, and calcium homeostasis	Promoting the assembly of Aβ into neurotoxic oligomers and fibrils	SARS-CoV-2	Promoting the emergence of COVID-19 neuropathological changes	[73, 74, 77]
*CD33*	Regulating immune cell function	Affecting microglia-mediated Aβ clearance	HBV; SARS-CoV-2	Mediating immunosuppression and immune escape	[78, 84, 85]
*SPI1*	Regulating microglia development, function and homeostasis	Activating microglia in response to neurotoxic ligand stimulation	EBV	Increasing the level of proinflammatory cytokines and promoting inflammation	[88, 90, 92]
*INPP5D*	Regulating the function of microglia	Inhibiting microglia proliferation, migration and phagocytosis	HCV; EBV	Inhibiting abnormal activation of inflammation	[93, 95, 96, 99]
*CD2AP*	Maintaining the integrity of intercellular connectivity	Maintaining synaptic structure and the integrity of the BBB	HCV; LCMV; CHIKV	Promoting microglia-mediated CNS inflammation	[56, 101-104, 167]
*PTK2B*	Neuronal plasticity and hippocampal related memory	Promoting tyrosine phosphorylation, tau accumulation, and Aβ-induced synaptic dysfunction and loss	HIV	Assisting virus entry, assembly, release, and activating the mitogen-activated protein kinase signaling	[108, 113-115]
*TREM2*	Promoting intracellular signal transduction	Promoting microglia proliferation to limit Aβ plaque growth	SARS-CoV-2; HIV; SIV; MHV-JHM	Inducing inflammation	[116-118, 122-126]
*PICALM*	Regulating endocytosis, autophagy and vesicular transport	Participating in the clearing of Aβ and tau	EV-A71; HSV; HHV-6A	Slowing down inflammatory processes	[127, 128, 133, 135-137]
*MEF2C*	CNS development and functional maintenance	Regulating neuronal migration and differentiation, synaptic development and remodeling, neuronal excitability, and neuroinflammation	HTLV-1; SIV	Preventing excessive inflammatory response	[45, 140, 144]

Abbreviations: Aβ, β-amyloid; AD, Alzheimer’s disease; *APOE*, apolipoprotein E; *APP*, amyloid precursor protein; BBB, blood-brain barrier; *BIN1*, bridging integrator 1; *CD2AP*, CD2-associated protein; *CD33*, cluster of differentiation 33; CHIKV, chikungunya virus; CNS, central nervous system; *CLU*, clusterin; COVID-19, corona virus disease 2019; cPLA2, cytosolic phospholipase A2; *CR1*, complement receptor type 1; EBV, epstein-barr virus; EV-A71, enterovirus A71; HBV, hepatitis B virus; HCV, hepatitis C virus; HHV-6A, human herpesvirus 6A; HIV, human immunodeficiency virus; HSV, herpes simplex virus; HTLV-1, human T-cell leukemia virus type 1; *INPP5D*, inositol polyphosphate-5-phosphatase; LCMV, lymphocytic choriomeningitis virus; *MEF2C*, myocyte enhancer factor 2C; MHV-JHM, JHM strain of mouse hepatitis virus; *PICALM*, Phosphatidyl inositol- binding clathrin assembly protein; *PILRA*, paired immunoglobulin-like type 2 receptor A; *PSEN1/2*, presenilin 1/2; *PTK2B*, Protein tyrosine kinase 2 beta; SARS-CoV-2, severe acute respiratory syndrome coronavirus 2; SIV, simian immunodeficiency virus; *SPI1*, salmonella pathogenicity island 1; *TREM2*, triggering receptor expressed on myeloid cells 2.

#### 1.1.2 Viral infection associated with APOE

*APOE* ε4 carriers were found to have higher levels of herpes simplex virus (HSV) -1 DNA in their cerebrospinal fluid than noncarriers, indicating a potential role for APOE in regulating the response to viral infection in the central nervous system (CNS), and that the expression and protein levels of APOE can be altered after viral infection [18].

*APOE* ε4 has the potential to impact the expression and functionality of specific cell surface receptors, including cluster of differentiation (CD) 4 and chemokine receptors, which play a crucial role in facilitating HIV entry into cells. This APOE isoform might also modify the lipid composition and microenvironment of the cell membrane, creating a more conducive environment for HIV-1 attachment and fusion. Moreover, *APOE* ε4 could modulate the immune response and contribute to neuroinflammation, potentially influencing the overall progression and outcomes of HIV infection [19] (Fig. 3A).

Additionally, it has been discovered that the receptor-binding domain of the SARS-CoV-2 spike (S) protein can interact with APOE and induce remarkable conformational changes in the protein, thus exploiting its metabolic pathway to facilitate viral entry into cells [20]. In addition, APOE can interact with the cell receptor angiotensin-converting enzyme 2 (ACE2) of SARS-CoV-2 through the zinc metalloproteinase domain of ACE2, hence interferes with ACE2/S protein-mediated viral entry. However, *APOE* ε4 has slightly weaker inhibitory effects than *APOE* ε3. This difference may be due to the more compact structure of *APOE* ε4, which results in less steric hindrance and competition with the binding of the S protein to ACE2. Clinically, the correlation between *APOE* ε4 carriers and COVID-19 patients suggests that *APOE* ε4 is associated with increased susceptibility to SARS-CoV-2 infection and elevated serum inflammatory factors [21] (Fig.3A).

### 1.2 Amyloid precursor protein (APP)

#### 1.2.1 Molecular information and biological function of APP

APP is a transmembrane protein mainly composed of a large extracellular domain, a single transmembrane domain, and a short cytoplasmic tail. The extracellular domain of APP contains multiple functional elements, including a copper-binding site, a heparin-binding domain, and a cystatin C binding domain [22]. The copper-binding site in the extracellular domain of APP can interact with metal ions, including copper and zinc. This interaction is suggested to play a role in regulating the physiological and pathological functions of APP. Copper binding to APP may influence its processing and promote the generation of toxic Aβ, which is implicated in AD [23]. The heparin-binding domain within the extracellular segment of APP interacts with heparan sulfate proteoglycans (HSPGs), which are components of the extracellular matrix in the brain. The interaction of APP with HSPGs can affect its trafficking, processing, and Aβ production, potentially contributing to AD pathology [24]. Regarding the signaling pathway involved in APP, the processing of APP is mediated by enzymes called secretases. α-secretase cleaves APP within the Aβ region, resulting in the release of a soluble fragment known as sAPPα. On the other hand, the cleavage of APP by β-secretase and subsequent γ-secretase generates Aβ, which can aggregate and form neurotoxic plaques in AD [25].

The expression of APP in neurons, particularly in neurons vulnerable to AD pathology, is closely related to AD onset. In particular, neurons in the hippocampus, neocortex, and other AD-affected regions show high levels of APP expression in *APP/tau* mice, which are transgenic mice that overexpress human mutant *APP* [26]. Increased expression of APP, particularly the isoform containing the Swedish mutation (*APPswe*), enhances Aβ production and deposition [27]. Studies using transgenic mouse models expressing human *APPswe* have shown accelerated Aβ pathology and cognitive impairments resembling AD [28]. Besides, emerging evidence suggests that the pathological product Aβ in turn can drive an inflammatory response by activating microglial and astrocytic cells, the main immunocompetent cells in the CNS, creating a vicious cycle in AD [29].

#### 1.2.2 Viral infection associated with APP

HSV infection has been shown to interfere with the processing of APP, leading to alterations in the production and accumulation of Aβ, contributing to the development or progression of AD neurodegenerative processes [30]. Meanwhile, HSV-1 infection enables the interaction between viral particles and cell membrane-bound APP through viral envelope glycoprotein E, leading to their cotransport into cells. This interaction not only affects the normal trafficking and distribution of APP with decreased transport velocity and abnormal distribution of APP as a result, but also contributes to accelerated viral invasion [31] (Fig. 3B).

A more complex interaction between APP and viral infection has been reported in COVID-19. The S protein of SARS-CoV-2 has been shown to inhibit the expression of APP in brain microvascular endothelial cells when attached to ACE2 on the cell surface [32]. Whereas, the following inflammatory responses mediated by tumor necrosis factor (TNF)-α, interferon-γ (IFN-γ), signal transducer and activator of transcription (STAT)-1, interleukin (IL)-1β, IL-6, and STAT-3 promote the production of APP [32].

Contradictory results have also been reported in Zika virus infection. APP is suggested to be a restriction factor that protects against Zika virus infection in mammalian brains, possibly by blocking viral entry into neurons and viral replication [33]. More intriguingly, the antimicrobial property of Aβ as a metabolic product of APP has also been reported. Specifically, Aβ oligomers bind to carbohydrates of the microbial cell wall *via* the VHHQKL domain, promoting new Aβ fibril formation and propagation. The resulting Aβ fibrils subsequently impair microbial adherence to host cells, trapping unattached microbes in an antiprotease-resistant amyloid network [34, 35].

### 1.3 Complement receptor type 1 (CR1)

#### 1.3.1 Molecular information and biological function of CR1

CR1, also known as CD35, is a cell surface glycoprotein involved in the regulation of the complement system. It plays a critical role in the innate immune response. The CR1 protein possesses a complex architecture comprising multiple complement control protein repeats, which play a crucial role in ligand binding and regulation of the complement system. CR1 binds to complement protein molecules C3b and C4b, participating in the degradation and clearance of immune complexes [36]. In the context of AD, studies have demonstrated altered expression of brain complement receptors, including CR1 [37], particularly in regions associated with Aβ deposition and neuroinflammation [38]. While the majority of complement proteins are synthesized in the liver, neuroglial cells and neurons in the central nervous system are also capable of producing multiple components of the complement system. In AD, the production of the complement cascade by these cells may be increased. A significant association between complement proteins and AD pathological hallmarks has been revealed, suggesting a potential detrimental impact of the complement system in the brain [39]. Furthermore, microglia exhibit an early phenotype of AD and become immunoreactive, serving as another risk marker indicating AD progression. The increased expression of CR1 in activated microglia has been associated with neuronal death in AD, as these cells produce a plethora of inflammatory cytokines such as TNF-α, and IL-1β, thereby exacerbating the inflammatory response [40].

#### 1.3.2 Viral infection associated with CR1

CR1 has been implicated in several aspects of the immune response to virus infection. CR1 plays a role in HIV replication in activated CD4+ T lymphocytes. Ligation of CR1 with cross-linked anti-receptor monoclonal antibodies or aggregated C3b resulted in a significant upregulation of viral gene transcription in CD4+ T lymphocytes, as well as the release of p24 (HIV core antigen) and reverse transcriptase activity [41]. Moreover, using immunostimulatory monoclonal antibodies against CR1 and CR3 or complement component 3 fragments, enhanced viral replication in monocytes has been obtained. This effect is accompanied by the translocation of nuclear factor kappa-light-chain-enhancer of activated B-cells (NF-κB) [42] (Fig. 3C).

### 1.4 Paired immunoglobulin-like type 2 receptor A (PILRA)

#### 1.4.1 Molecular information and biological function of PILRA

PILRA is a type I transmembrane glycoprotein that belongs to the paired immunoglobulin-like receptor family. There is evidence suggesting that genetic variations in the *PILRA* gene may be related to AD onset due to its role in modulating microglial activation [43]. PILRA is an immunoglobulin receptor that recognizes specific O-glycosylated proteins and is expressed on various innate immune cell types including microglia. *PILRA* G78 (rs1859788), a common missense variant of PILRA, is the causal allele for AD [44]. A study revealed a significant positive correlation between the levels of *PILRA* gene expression and the density of both Aβ plaques and phosphorylated tau (p-tau) protein in microglial cell transcripts [45]. A large-scale GWAS analysis has provided evidence that PILRA is involved in microglia-mediated neuroinflammation and is associated with an increased risk of late-onset AD [46] (Fig. 3D). Carrying the A allele of the *PILRA* rs1859788 gene, along with the C allele of the *FCGRIIB* gene, can triple the risk of developing AD [47].

#### 1.4.2 Viral infection associated with PILRA

PILRA acts as an entry receptor for HSV-1 by binding to viral glycoprotein B, which is required for fusion between the viral envelope and the host cell membrane. The interaction between PILRA and glycoprotein B enables the penetration and entry of HSV-1 into the host cell [44]. In addition to HSV-1, PILRA has been shown to mediate the invasion of HSV-2 and its pseudorabies virus [48, 49] (Fig. 3D).

### 1.5 Clusterin (CLU)

#### 1.5.1 Molecular information and biological function of CLU

The *CLU* gene encoding clusterin is located on chromosome 8p21-p12 in humans. This gene spans over 16 kilobases and contains 9 exons, which are segments of the gene containing information for protein synthesis [50]. CLU, also known as apolipoprotein J, is a glycoprotein that exists as a heterodimeric protein composed of two subunits, α and β, that are linked by disulfide bonds [51]. Its structure comprises a series of alpha-helices and beta-sheets, with N-glycosylation sites in both subunits [52]. CLU can interact with different ligands, such as lipids, complement components, and the Aβ peptide, and is involved in the process of lipid metabolism, apoptotic and inflammatory pathways, and amyloid deposition in the brain [53]. The absence of CLU in neurons does not manifest neurodegeneration in response to Aβ, highlighting the essential role of CLU as an effector of Aβ toxicity [54] (Fig. 3E). Meanwhile, elevated CLU has also been shown to accelerate the spreading of tau aggregates in AD patients, suggesting that CLU can accelerate AD progression [55, 56].

#### 1.5.2 Viral infection associated with CLU

It was revealed that plasma CLU could interact with nonstructural protein 1 of dengue virus, resulting in the activation of the complement system and subsequent occurrence of plasma leakage, thereby promoting the progression of dengue fever [57] (Fig. 3E).

HSV-1 infection has emerged as a potential risk factor for AD. Interestingly, CLU resembles the mannose-6-phosphate receptor, a crucial component exploited by HSV for cellular invasion and intracellular transportation. Hence, it is conceivable that CLU is actively engaged in the life cycle of HSV, thereby influencing the intricate interplay between HSV and pivotal proteins implicated in the intricate processing of APP and Aβ [58] (Fig. 3E).

Besides, in brain-like organoids infected with SARS-CoV-2, the main affected cells are choroid plexus epithelial cells with abundant CLU expression. SARS-CoV-2 infection impairs the choroid plexus epithelium, leading to blood-brain cerebrospinal fluid barrier leakage, which fails to prevent the entry of pathogens, immune cells, and cytokines into the brain. This intriguing finding sheds light on the intricate association between CLU and the invasion of the nervous system by SARS-CoV-2 [59, 60] (Fig. 3E).

### 1.6 Bridging integrator 1 (BIN1)

#### 1.6.1 Molecular information and biological function of BIN1

BIN1, encoded by the *BIN1* gene, is a widely expressed nucleocytoplasmic adaptor protein that is part of the BIN1/Amphiphysin/Rvs167 family. BIN1 contains several domains, including an N-BAR (BIN/Amphiphysin/Rvs) domain, a src homology (SH) 3 domain, and a myc-like DNA-binding domain [61]. Functionally, BIN1 is engaged in membrane remodeling and endocytosis [61]. More specifically, in clathrin-mediated endocytosis, BIN1 interacts with the adaptor protein 2 complex and promotes invagination of the plasma membrane to initiate endocytic events [62]. In the nervous system, BIN1 is primarily expressed in different types of nerve cells, including neurons and glial cells [63]. Within neurons, BIN1 is found in both presynaptic and postsynaptic compartments, where it plays a role in synaptic vesicle endocytosis and recycling [64]. The risk variant of *BIN1* associated with AD is believed to modulate its expression levels or protein isoforms, impacting the protein’s function in neuronal cells and potentially disrupting synaptic processes [65]. BIN1 may promote tau aggregation by modulating tau phosphorylation and neuronal cytoskeletal dynamics, thereby indirectly triggering neuroinflammation and ultimately promoting neurodegeneration [62, 66].

#### 1.6.2 Viral infection associated with BIN1

It has been proposed that nonstructural protein 5A (NS5A), a hepatitis C virus (HCV) component that is crucial for viral replication and assembly, can disrupt the interaction between BIN1 and the c-Myc protein by interacting with BIN1 through the highly conserved polyproline SH3 binding motif located in the D2 and D3 regions of NS5A [67]. The interaction between NS5A and BIN1 implies that HCV infection may interfere with the tumor-suppressive function of BIN1, potentially promoting the growth and proliferation of cancer cells [68] (Fig. 3F).

In the context of SARS-CoV-2 infection, BIN1 has been shown to hinder viral infection through the following mechanisms: i) BIN1 may disrupt viral infection by interacting with SARS-CoV-2 NSP1; and ii) by binding to NSP3, BIN1 could potentially interfere with the conformational changes of this viral protein, therefore limiting the release of newly synthesized viral RNA [69].

### 1.7 Presenilin (PSEN) 1 and PSEN2

#### 1.7.1 Molecular information and biological function of PSEN1/2

PSEN1 and PSEN2 are multipass membrane proteins that function as the catalytic subunit of the γ-secretase complex, which is composed of at least four different protein components: anterior pharynx-defective 1, protein enhancer of gamma-secretase 2, nicastrin, and PSEN [70]. PSEN1 and PSEN2 both accommodate nine transmembrane domains and two large hydrophilic loops located on the cytosolic side of the membrane [71]. The active site of γ-secretase, which is located within the lipid bilayer, consists of two key aspartate residues of PSENs [72]. Functionally, PSEN1/2 proteins perform critical roles in synaptic activities, cell survival pathways and calcium homeostasis [73]. Reports suggest that mutations in PSEN1/2 are associated with early-onset familial AD [73]. These mutations appear to cause a gain of function, driving an increase in the ratio of longer, more aggregation-prone isoforms of Aβ42, thereby promoting the assembly of Aβ into neurotoxic oligomers and fibrils. Besides, through nonamyloidogenic activities, PSEN1/2 mutations maintain their role in exacerbated neurodegeneration by mechanisms related to disrupted calcium homeostasis and enhanced ER-stress [74]. In the context of *APP/PS1* transgenic mice, astrocytes of this AD model are involved in the activation of NF-κB transcriptional activity, leading to the production of inflammatory factors and hence a loss of their neuroprotective function [75].

#### 1.7.2 Viral infection associated with PSEN1/2

ACE2 is the cell surface receptor that SARS-CoV-2 uses for invasion. After binding with the S protein of SARS-CoV-2, ACE2 is hydrolyzed by transmembrane protease serine 2, leading to cleavage of the extracellular domain of ACE2. The γ-secretase targets intracellular ACE2 for protein hydrolysis, releasing soluble ACE2 and establishing new ACE2 transport pathways [76]. It has been reported that in *APP/PS1* transgenic mice, the S2 subunit of the SARS-CoV-2 S protein can interact with the γ-secretase complex, enhancing γ-secretase cleavage of APP to accelerate the production of Aβ, thereby promoting the emergence of COVID-19 neuropathological changes [77] (Fig. 3G).

### 1.8 Cluster of differentiation 33 (CD33)

#### 1.8.1 Molecular information and biological function of CD33

CD33, also known as sialic acid-binding immunoglobulin-like lectin (Siglec)-3, is an inhibitory immune receptor and type I transmembrane protein involved in the regulation of innate and adaptive immune systems by recognizing glycan ligands [78]. As a cell surface transmembrane receptor, CD33 consists of two extracellular immunoglobulin domains, including sialic acid binding sites. Its cytoplasmic domain has an immune receptor tyrosine-based inhibitory motif based on the immune receptor tyrosine and sends a negative signal *via* recruitment of tyrosine phosphatase [79]. Unlike Siglecs, which are conserved in mammals, such as sialoadhesin, Siglec-2, and Siglec-15, CD33 is evolutionarily diverse in mammals [79].

In the brain, CD33 is expressed in microglia and has received much attention as one of the genes strongly associated with the risk of developing AD. In postmortem brain samples from AD patients, CD33 mRNA and protein levels were elevated, contributing to Aβ pathology by affecting microglia-mediated Aβ clearance [80]. However, in *CD33*-knockout *APP/PS1* mice and *5xFAD* mice, amyloid plaque reduction and cognitive improvement were observed [80, 81]. Moreover, in microglia derived from *CD33*-knockout mice, the uptake of Aβ was promoted, and the inhibition of Aβ uptake was inhibited when CD33 was elevated [80] (Fig. 3H). In addition, the protective allele of the *CD33* single-nucleotide polymorphism (SNP) rs3865444 is associated with decreased levels of insoluble Aβ42 in AD brains [80].

#### 1.8.2 Viral infection associated with CD33

CD33 is one of the key markers used to identify human myeloid-derived suppressor cells (MDSCs), which are important mediators of immune escape and immunosuppression [82]. The frequency of the *CD33* rs3865444 CC allele increases in patients with AD, which subsequently upregulates microglial CD33 expression, hence increasing the immunosuppressive CD33 MDSC population, leading to enhanced susceptibility and more severe infection [83, 84]. For instance, the variability SARS-CoV-2 infection is remarkable, ranging from asymptomatic to death, and the immunosuppressive CD33 MDSC populations are increased in all groups at risk of severe COVID-19 infection [84] (Fig. 3H).

CD33 plays an important role in the progression of hepatitis B virus (HBV) infection. HBV binds to and then activates CD33 *via* alpha 2,6-biantennary sialoglycans of the surface antigen. By quantifying the frequency of cells expressing CD33 and HBV surface antigen (HBsAg) using peripheral blood mononuclear cells isolated from patients with chronic hepatitis B, 56.5% of CD33-positive cells were found to be bound to HBsAg and almost all HBsAg-positive cells were bound to CD33 [85]. Specifically, upon binding with HBV, CD33 attenuates cell activation and induces immunosuppression by recruiting protein tyrosine phosphatase 1/2 (Fig. 3H). In contrast, blocking CD33 can enhance immune regulation mediated by the Toll-like receptor 7 agonist GS-9620 and increase the production of TNF-α and IL-6 [85].

### 1.9 Salmonella pathogenicity island 1 (SPI1)

#### 1.9.1 Molecular information and biological function of *SPI1*

*SPI1* is a 40-kb island consisting of a type III secretion system [86]. The transcription factor PU.1, encoded by *SPI1*, is a major regulator of microglial development, function and homeostasis [87]. PU.1 is expressed in microglia in the CNS and is associated with susceptibility to AD. Neurotoxic ligands such as Aβ42 and p-tau mediate the activation of stimulus-dependent transcription factors, which in turn correlate with the enhancer sequence of PU.1, resulting in higher expression of PU.1. The induced expression of PU.1 increases the activation of microglia and the levels of the proinflammatory cytokines TNF-α, C-X-C motif chemokine ligand (CXCL) 2, CXCL10, C-C motif chemokine ligand (CCL) 5, CCL12, IL-6 and TREM1 and aggravates neuroinflammation [88] (Fig. 3I).

In European populations, several SNPs have been identified as protective factors for AD, namely rs3740688, rs1057233, and rs78245530 [89, 90]. However, in the Chinese population, only rs3740688 showed a protective effect. The protective *SPI1* haplotypes identified were β (labeled by rs1057233 and rs3740688) and γ (labeled by rs3740688 and rs78245530) [90]. Haplotypes β and γ are associated with decreased expression levels of the *SPI1* gene in blood and brain tissue, respectively, and their regulatory effects are involved in immune and neuronal functional pathways [90].

#### 1.9.2 Viral infection associated with *SPI1*

The Epstein-Barr virus (EBV) has the ability to cause sustained proliferation of lymphoblastic cell lines [91, 92]. Chromatin immunoprecipitation-sequencing has revealed that the EBV nuclear antigen 3C possesses a *SPI1* binding domain, thereby leveraging an intrinsic B-cell program to drive cellular immunity [92] (Fig. 3I). In addition, EBV-associated B lymphomas and Hodgkin disease are more prevalent in T-cell immunodeficient individuals, indirectly contributing to death in HIV-infected patients [92].

### 1.10 Inositol polyphosphate-5-phosphatase (INPP5D)

#### 1.10.1 Molecular information and biological function of *INPP5D*

The SH2 domain-containing inositol phosphatase-1 (SHIP1) protein encoded by *INPP5D* has two main functional domains, the SH2 binding domain and phosphatase domain [93]. The former is responsible for its migration to the cell membrane, where it colocalizes with activated TREM2 and its coreceptor DNAX activation protein (DAP) 12. The latter is responsible for the dephosphorylation of secondary messengers, thereby inhibiting signal transduction [93]. SHIP1 negatively regulates microglial TREM2 signaling *via* the phosphatidylinositol 3 kinase (PI3K) pathway [94].

*INPP5D* expression is almost exclusively restricted to microglia in the CNS, inhibiting microglial proliferation, migration and phagocytosis. As a risk factor for AD, increased *INPP5D* expression is associated with increased plaque deposition, and knockdown of *INPP5D*, thereby reducing SHIP1, has a protective effect on AD (Fig. 3J). It has been shown that in *INPP5D* haplodeficient and *INPP5D*-knockout mice, plaque associated microglia numbers are increased, their phagocytosis of Aβ is enhanced, and cognitive performance is improved [95, 96]. In addition, two SNPs of *INPP5D* are closely related to AD risk, with rs35349669 being associated with increased AD risk while rs10933431 is associated with reduced AD risk [94].

#### 1.10.2 Viral infection associated with *INPP5D*

Persistent inflammation is a major determinant in the progression from hepatic fibrosis to cirrhosis, which is regulated by the innate immune system through toll-like receptor-dependent signaling [97]. As an inhibitor of this signaling pathway, *INPP5D* plays a significant role in the regulation of liver fibrosis due to chronic HCV infection. Using microarray analysis and quantitative reverse transcription polymerase chain reaction, studies with peripheral whole blood samples of HCV patients have shown a significant negative correlation between *INPP5D* gene expression and the degree of fibrosis [98].

In EBV infection, *INPP5D* is the target of EBV miRNA related to B-cell receptor signal transduction, and the interaction of the two attenuates the downstream activation of B-cell receptor-initiated NF-kB and activator protein 1-dependent transcription. In this context, *INPP5D* functions to help cells maintain a latent infection state to avoid abnormal reactivation [99] (Fig. 3J).

### 1.11 CD2-associated protein (CD2AP)

#### 1.11.1 Molecular information and biological function of *CD2AP*

The *CD2AP* gene is located on chromosome 6 and contains 18 exons. The encoded CD2AP protein is composed of 639 amino acids spanning across three SH3 domains, a proline-rich domain, and an actin-binding region, the latter of which promotes the integrity of intercellular connectivity through resistance to mechanical stress [100]. Functionally, CD2AP can indirectly promote microglia-mediated CNS inflammation. By forming a complex with SHIP1, CD2AP subsequently inhibits Casitas B lineage lymphoma [101], which is an E3 ubiquitin ligase associated with the regulation of immune cell activities and inhibition of microglia-mediated inflammation through the PI3K/Akt/NF-κB pathway [102]. Additionally, CD2AP is involved in maintaining blood-brain barrier (BBB) integrity. In *CD2AP*-knockout mice, reduced BBB integrity has been reported [56].

#### 1.11.2 Viral infection associated with CD2AP

Chronic lymphocytic choriomeningitis virus (LCMV) infection causes dysregulation of CD8 T-cells. CD4 T-cells express cytokines and costimulatory molecules that support a sustained CD8 T-cell response and enhance the production of protective antibodies by germinal center B-cells [103]. CD2AP inactivation promotes CD4 T-cell differentiation towards the follicular helper lineage, leading to enhanced control of viral infection by augmented germinal center response in chronic LCMV infection [103].

Chikungunya virus (CHIKV) can cause arthritis and arthralgia. Quantitative proteomic studies have found that the C-terminal hypervariable domain of viral replicase proteins can bind with CD2AP, leading to reduced CHIKV replication [104].

HCV infection can lead to steatosis. CD2AP upregulation has been detected in both HCV-infected mice and human livers. This upregulation is closely related to HCV reproduction and liver steatosis [105]. CD2AP has two roles in HCV pathogenesis. First, it binds with NS5A and participates in NS5A transport to lipid droplets (LDs) to facilitate viral assembly. Second, it disrupts insulin signaling and increases LDs accumulation through the IRS1/Akt/AMPK/HSL signaling axis to adapt to viral assembly [105] (Fig. 3K).

### 1.12 Protein tyrosine kinase 2 beta (PTK2B)

#### 1.12.1 Molecular information and biological function of PTK2B

Proline-rich tyrosine kinase 2 (Pyk2), encoded by *PTK2B*, is a 110 kDa protein containing 1009 amino acids. It contains several well-defined domains, including an N-terminal domain, a central tyrosine kinase domain, and a C-terminal focal adhesion targeting domain [106]. Pyk2 is highly expressed in the central nervous system and exists in the cytoplasm and postsynaptic density. It plays an important role in neuronal plasticity and hippocampal-related memory, and its dysregulation leads to cognitive deficits in neurodegenerative diseases. This enzyme is involved in calcium-induced ion channel regulation and activation of the mitogen-activated protein kinase signaling pathway in response to various stimuli [107, 108].

The most interesting function of Pyk2 in AD is its regulatory role in tau phosphorylation. Glycogen synthase kinase 3, a known tau phosphorylated kinase, is activated by Pyk2 [109]. Pyk2 is colocalized with hyperphosphorylated and oligomeric tau, and its overexpression showed increased tyrosine phosphorylation and tau accumulation [108, 110] (Fig. 3L). However, *PTK2B* deficiency did not show beneficial effects, as it also leads to increased tau phosphorylation and memory impairment [111]. In addition to tau accumulation, Pyk2 mediates Aβ-induced synaptic dysfunction and loss in AD [112, 113].

#### 1.12.2 Viral infection associated with PTK2B

HIV-1 glycoprotein 120 binds to the T-cell receptor CD4 and then induces Pyk2 phosphorylation and activation. The activation of tyrosine phosphorylation signaling is required for viral entry, actin remodeling, and nuclear translocation, thereby promoting viral infection [114]. For instance, HIV-1 assembly occurs in the virus-containing compartment of macrophages, where Pyk2 regulates the adherence-like shell and anchors the actin cytoskeleton to the virus-containing compartment to promote viral particle release [115] (Fig. 3L). Consistently, inhibition of Pyk2 activation was conducive to inhibition of virus entry, assembly and release of virus particles [114, 115].

### 1.13 Triggering receptor expressed on myeloid cells 2 (TREM2)

#### 1.13.1 Molecular information and biological function of TREM2

TREM2 is a transmembrane receptor of the immunoglobulin superfamily [116]. TREM2 binds to DAP12 or DAP10 to form a TREM2-DAP12 or TREM2-DAP10 heterodimer [93]. A variety of ligands can bind to TREM2, including lipoproteins, phospholipids, DNA, apoptotic cells, and bacterial products [117]. When TREM2-ligand interactions occur, spleen tyrosine kinase and PI3K are recruited to promote intracellular signal transduction [116].

TREM2 plays a critical role in the activation of microglia and macrophages, and may act as a downstream mediator of CD33 in regulating microglial pathologies in AD [116]. TREM2 promotes microglial proliferation and survival, which exerts neuroprotective effects by creating a physical barrier around Aβ aggregates to limit plaque growth [81, 118] (Fig. 3M). Genetic studies based on European-American and African-American populations reveal that there are multiple TREM2 genetic variants that increase AD risk. With the R47H variant being the most relevant, other low-frequency coding variants of *TREM2*, such as R62H, R52H, T66M, R136W, R136Q, H157Y, W191X, E202D, H215Q and T223I, have been exclusively found in AD cases [119]. The rare missense mutation (rs75932628-T) in *TRME2*, which was predicted to result in R47H substitution, was found to confer AD risk (odds ratio, 2.92) with an effect size that is similar to that of *APOE* ε4 [120]. In experimental models, knocking out *TREM2* in *PS2APP* and *5xFAD* AD model mice resulted in a more severe plaque load, neuroinflammation, and neurodegeneration [81, 121].

#### 1.13.2 Viral infection associated with TREM2

TREM2 acts as a triggering receptor in SARS-CoV-2 infection and is therefore associated with infection susceptibility and severity. The SARS-CoV-2 membrane protein binds to TREM2, which activates inflammation by enhancing the production of IFN-γ, TNF-α, IL-4, IL-6, IL-10, IL-1β, and other proinflammatory factors (Fig. 3M). Therefore, TREM2 promotes virus clearance and protects pulmonary function [122, 123]. A study in rhesus macaques showed that SARS-CoV-2 infection caused rapid recruitment of TREM2 macrophage subsets, driving airway inflammation [90]. Since an excessive inflammatory response can induce cytokine storms, this also explains why TREM2 is significantly elevated in patients with severe COVID-19 [123].

HIV-associated neuroinflammation is also closely connected with TREM2. High levels of soluble TREM2 (sTREM2) in the cerebrospinal fluid have been shown to be a common feature of untreated HIV infections, which increase with CD4+T-cell loss and reach the highest level in HIV-associated dementia [124]. In a simian immunodeficiency virus (SIV) model of HIV in macaques, the level of TREM2 in the frontal cortex was much higher in the SIV-infected group than in uninfected controls, supporting the role of TREM2 in the pathogenesis of HIV CNS disease [125].

Using a neurotropic, attenuated JHM strain of mouse hepatitis virus (MHV-JHM) infection model of acute encephalomyelitis, which resolves into a persistent infection associated with demyelination, a recent study showed that *TREM2* deficiency does not affect viral control or establishment of persistence, but is essential in the clearance of damaged myelin induced by viral infection [126]. This result highlights the importance of TREM2-promoted phagocytic activities in the removal of viral infection-induced toxins.

### 1.14 Phosphatidyl inositol-binding clathrin assembly protein (PICALM)

#### 1.14.1 Molecular information and biological function of PICALM

*PICALM* is located on chromosome 11, and the encoding PICALM protein consists of an N-terminal of the AP180 N-terminal homology domain binding phos-phatidylinositol 4,5 diphosphate and a C-terminal clathrin adapter domain [127]. PICALM is generally expressed in all tissue types, prominently in neurons, and is nonselectively distributed in presynaptic and postsynaptic structures. Functionally, PICALM is involved in clathrin-mediated endocytosis and autophagy, contributing to the transport of intracellular proteins and lipids, as well as the clearance of damaged cell structures and biomacromolecules [127-130].

The *PICALM* was a major genetic susceptibility locus for AD, which has been ranking second to *APOE* in a GWAS, and an interaction between *PICALM* and *APOE* has been reported [131]. Functionally, the expression of *PICALM* is beneficial for AD. In induced pluripotent stem cells-derived human astrocytes, *PICALM* rescues endocytic defects caused by *APOE* ε4 [132]. Moreover, PICALM is involved in the clearance of Aβ and p-tau through endocytosis. [127, 133] (Fig. 3N). Besides, the levels of reactive oxygen species and peroxides are generally increased in AD. To delay neurotoxicity induced by these chemicals, LDs in glial cells are formed to encapsulate peroxides, and PICALM has been shown to contribute to the formation of neuroprotective LDs [134]. Likewise, reduced PICALM expression increases the risk of AD. Heterozygous *PICALM*-knockout *Tg30* mice exhibit abnormally higher levels of p-tau, neurofibrillary tangles and autophagy markers in the brain than *Tg30* mice [133].

#### 1.14.2 Viral infection associated with PICALM

Enterovirus A71 (EV-A71) causes hand, foot, and mouth disease, which is associated with neurological complications. PICALM is an intermediate mediator regulating the associated infection response. Exosomes containing host cell miRNAs can regulate cellular responses during viral infection. miR-155, which is significantly enriched in exosomes after EV-A71 infection, could effectively reduce the infection severity by repressing *PICALM* [135] (Fig. 3N).

Moreover, a GWAS suggested that the genetic characteristics of *PICALM* potentially influenced brain susceptibility to the HSV family during aging [136]. Other studies have suggested that PICALM is related to the life cycle of HSV and is involved in the entry and transport of virions within the host cell [58] (Fig. 3N).

Additionally, by integrating genomic, transcriptomic, proteomic, and histopathological data, one study reported an increase in human herpesvirus 6A (HHV-6A) in the brains of patients with late-onset AD, which is associated with the interaction between HHV-6A and PICALM [137] (Fig. 3N).

### 1.15 Myocyte enhancer factor (MEF) 2C

#### 1.15.1 Molecular information and biological function of MEF2C

MEF2C is an important member of the MEF2 family and consists of five core domains: MCM1-Agamous-Deficiens-serum response factor domain, MEF2 domain, transcriptional activation domains 1 and 2, and nuclear localization signal [138]. MEF2C is essential for nervous system development and functional maintenance, and is involved in the regulation of neuronal migration and differentiation, synaptic development and remodeling, neuronal excitability, and neuroinflammation [139].

MEF2C is mainly expressed in astrocytes and microglia in the brain. It appears to play a neuroprotective role in AD by preventing excessive inflammation and maintaining environmental homeostasis [139] (Fig. 3O). Relatively low levels of MEF2C thus contribute to the occurrence and development of AD. In *MEF2C*-knockout mice, the microglial response is more intense, and CCL2, CCL5, IL-1β, and TNF expressions are significantly increased; these changes result in more severe cognitive impairment [140]. *MEF2C* mRNA levels are lower in white blood cells of AD patients and reportedly correlate with AD Assessment Scale and Mini-Mental State Examination scores [141]. Paradoxically, however, single-nucleus RNA sequencing of postmortem brain astrocytes in AD patients has demonstrated upregulation of MEF2C, which is significantly correlated with Aβ and p-tau [45]. Differences among different ethnic groups have also been revealed in studies of *MEF2C* SNPs. For example, the *MEF2C* polymorphism rs190982 has been reported as a protective factor against AD in a Caucasian population but as a risk factor for AD in a population from Taiwan, China [142, 143].

#### 1.15.2 Viral infection associated with MEF2C

Human T-cell leukemia virus type 1 (HTLV-1) is a retrovirus that causes adult T-cell leukemia and lymphoma (ATLL). MEF2C is overexpressed in a variety of ATLL cell lines and patients with acute ATLL. Inhibition of MEF2C protein leads to cytotoxicity of ATLL cells *in vitro* and a reduced proviral load *in vivo* [144] (Fig. 3O).

SIV is another virus that is associated with MEF2C. Infection with SIV results in damaged myoblast differentiation, with concomitant reductions in muscle-specific miRNA-206 and MEF2C expression levels [145].

## 2. Genetic risk factors for AD that are weakly or not associated with viral infection

In addition to the AD risk genes that are associated with viral susceptibility, several other genes have been identified as AD risk factors but are weakly correlated with or have not been clearly demonstrated to correlate with viral susceptibility. Genes that may impact viral infections are *ABCA7* and *HLA-DRB5/DRB1. ABCA7* is highly associated with brain amyloidosis. Positron emission tomography has revealed that the *ABCA7* SNP rs3752246 is significantly correlated with Aβ deposition (χ^2^ = 8.38, corrected *P* < 0.001) [146], and the rs3764650 and rs200538373 SNPs increase the risk of AD to 1.23 and 1.91 times, respectively [147]. Furthermore, results of a GWAS suggest that *ABCA7* may affect the brain’s ability to cope with herpesvirus invasion [136]. *HLA-DRB5/DRB1* is mainly related to the immune response in AD-related neuroinflammation, and the *HLA-DRB5/DRB1* SNP rs9271192 is protective against late-onset AD [148]. In addition, *HLA-DRB5/DRB1* has been proposed to affect viral infection susceptibility [149, 150].

Aside from the two aforementioned genes, many other AD risk-related genes—including *RIN3*, *CASS4*, *SORL1*, *EPHA1*, and *MS4A*—have not yet been investigated in viral infections. In AD, *RIN3* upregulation promotes APP cleavage and increases phosphorylated tau levels [151, 152]. *CASS4* is a toxic modifier of tau, a pathological hallmark of AD [110]. *SORL1* is highly expressed in brain neurons, and its mutation promotes Aβ deposition and induces vasculitis or perivasculitis [153, 154], thus increasing the risk of late-onset AD [155]. *EPHA1* is involved in cell-cell interactions and is crucial for development, tissue homeostasis, and synaptic plasticity [156]. The protein encoded by *MS4A* is co-localized with TREM2 on plasma membrane lipid rafts, and overexpression of *MS4A* increases sTREM2 concentration and is associated with both a reduced risk and delayed onset of AD [157]. It is therefore of great interest to further investigate whether and how these genes exert their biofunctions in the context of viral infections.

## 3. BBB abnormalities in AD are associated with viral infection

The BBB is a complex biological structure composed of tightly linked endothelial cells, pericytes, astrocytes, and the basal lamina. The main functions of the BBB are to protect the brain from harmful substances in the blood, transport necessary nutrients, and regulate ion balance and immune surveillance in the CNS. In AD, both the integrity and function of the BBB are compromised; this is manifested by leaky endothelial junctions and the altered expression of various transport proteins in endothelial cells [158]. Neuronal impairment occurs concomitantly with the increased permeability of the BBB and disruptions in select substance transport mechanisms; together, this allows peripheral Aβ to access the brain and consequently contributes to AD progression [159, 160]. Viral infection, either as a direct viral attack or *via* indirect immune overactivation incited by invading virions, is a main cause of BBB disruption [161]. In the context of AD, virus-induced inflammation exacerbates pre-existing BBB damage and accelerates neural damage. The subsequent elevated infiltration of activated immune cells, cytokines, and possibly the virus itself into the CNS may further exacerbate the neuroinflammatory state of AD [162]. Furthermore, some neurological manifestations of SARS-CoV-2 infection, such as encephalitis, meningitis, or ischemic strokes, may indirectly contribute to the cognitive decline seen in AD patients [163].

Several genetic risk factors of AD have also been implicated in BBB impairment. For example, *APOE* ε4 carriers have reduced tight junction protein expression as well as altered structures of endothelial cells associated with BBB function [164] (Fig. 3A). It has also been hypothesized that CLU protects against the detrimental effects of Aβ on the BBB by mediating low-density lipoprotein receptor related protein 2 [165]. Furthermore, previous study has suggested that TREM2 signaling is essential for microglial responses to neuronal damage as well as for Aβ clearance. Functionally reduced TREM2 activity is responsible for a hyper-reactive immune response, thus promoting neuroinflammation and BBB disruption [166]. In addition, CD2AP helps to maintain BBB integrity, the impairment of which is correlated with abnormal Aβ metabolism, enhanced tau-induced neurotoxicity, altered neurite structure, and weakened BBB integrity [167].

## 4. Discussion

Dozens of gene loci are reportedly associated with AD; these genetic variants may result in the upregulation, downregulation, or specific isoform or mutant forms of gene products. For example, human APOE is expressed in three genetic isoforms: APOE2, APOE3, and APOE4. Of these isoforms, APOE4 with *APOE* ε4 is a risk factor for AD, whereas APOE2 carrying *APOE* ε2ε2 or *APOE* ε2ε3 plays a protective role [14]. The existence of SNPs in genes can not only change the types of amino acids in the resulting proteins (and consequently determine individual phenotypes), but can also affect disease susceptibility. For example, CD33 upregulation is associated with Aβ accumulation and an increased risk of developing AD, whereas the *CD33* SNP rs3865444 is associated with reduced Aβ levels [80]. Moreover, the *INPP5D* SNP rs35349669 confers an increased risk of AD, whereas the rs10933431 SNP is associated with decreased AD risk [94]. Furthermore, the effect of SNPs can vary across different populations. For example, the *MEF2C* SNP rs190982 is a protective factor in the Caucasian population but a risk factor in the Taiwanese population [142, 143]. Detailed investigations into the effects of specific gene loci are therefore required to further clarify the functionalities of genetic variants in the susceptibility, treatment, and prognosis of AD.

Many viral infections are more likely to occur in AD patients; these infections generally result in more serious clinical consequences and the further aggravation of neurological impairments. Of the known AD risk genes, some are relevant to the increased susceptibility of viral infections *via* processes related to viral life cycles, including viral entry, replication, assembly, and release, as well as other mechanisms, such as phagocytosis, immune regulation, and BBB changes [42, 48, 67, 84, 168, 169]. In turn, viral infections may confer a higher risk for cognitive decline and expedite the progression of AD by affecting Aβ accumulation, tau phosphorylation, synaptic structure and function, neuronal apoptosis, oxidative stress, and inflammation [17, 29, 30, 45, 167, 170]. For example, HSV-1 enters host brain and remains in a dormant state during middle age; however, following stress or inflammatory stimulations, HSV-1 can be reactivated, leading to a series of detrimental effects. This is particularly true in individuals with *APOE* ε4 genotype, in whom the severity of such damages may be exacerbated [171]. Similarly, Zika virus accelerates AD-related phenotypes, such as the production of Aβ and p-tau, in cerebral organoids by inducing endoplasmic reticulum stress [172]. In addition to the viruses discussed in the present review, other viral infections are associated with AD but lack evidence of a genetic background. For example, the risk of dementia in patients with genital warts caused by human Papillomavirus infection is 1.485 times higher than that of non-infected people [173]. Moreover, dementia risk in patients with herpes zoster is 2.83 times higher than that of uninfected people [174]. Nonetheless, further explorations are required to determine whether and how these viruses correlate with AD risk genes.

Some of the aforementioned viruses are more relevant to certain AD risk genes. In particular, SARS-CoV-2 infection could be driven by *APOE*, *APP*, *BIN1*, *PSEN1/2*, *CD33*, and *TREM2*; EBV infection is associated with *SPI1*, *INPP5D*, and *MEF2C*; HIV infection is linked to *APOE* and *TREM2*; HCV infection is correlated with *BIN1*, *INPP5D*, and *CD2AP*; and HSV-1 infection has associations with *APOE*, *APP*, *PILRA*, *CLU*, and *PICALM*. However, different genes may respond differently to a single viral infection, and some genes may act differently at different stages of infection. For example, BIN1 binds to SARS-CoV-2 to block viral infection, whereas CD33 increases SARS-CoV-2 susceptibility and infection severity because of its immunosuppressive effect [83, 175]. TREM2 acts as a trigger receptor and inflammatory modulator in SARS-CoV-2 infection; this activates the inflammatory pathway to promote viral clearance in the early stage of infection. However, it may induce inflammatory storms in the later stages, which can result in worse clinical outcomes [122, 123].

More intriguingly, upregulated levels of some genes are associated with increased AD risk but protect against viral infections, and *vice versa*. For example, upregulated levels of *APP*, *BIN1*, and *INPP5D* are connected with increases AD risks but play inhibitory roles in some viral infections. Specifically, increased APP expression promotes Aβ accumulation but can interfere with Zika entry and replication [27, 33]. Similarly, BIN1 enhances Aβ and tau aggregation and disrupts synaptic processes, but impairs the structure and function of SARS-CoV-2 [62, 65, 66, 176]. Additionally, INPP5D inhibits microglial Aβ phagocytosis but mitigates HCV-associated liver fibrosis and prevents abnormal cell activation during EBV infection [98, 99, 177]. In contrast, the downregulation of *TREM2* and *PICALM* gene products increases AD risks but may promote viral infections. Specifically, TREM2 promotes Aβ phagocytosis but triggers HIV- and SIV-induced neuroinflammation [118, 124, 125]. Moreover, PICALM is involved in Aβ and tau clearance but mediates HSV entry and transport in host cells [58, 178]. Together, these findings indicate that the same gene may have broad and multifaceted features, participating in different stages of AD pathology and virus infection. It is therefore difficult to draw simple conclusions about whether an AD risk gene is a risk gene for or a protective factor against viral infection.

Given the complexity of many of the genes involved in AD development, certain genes have emerged as important biomarkers for the early prediction of AD. The most well-established genetic risk factor for AD, *APOE*, is one such biomarker. When magnetic resonance imaging is combined with *APOE* gene sequencing, the accuracy of predicting AD onset is greater than 80% [179]. Another potential peripheral diagnostic and prognostic biomarker is TREM2, a receptor that promotes immune cell function and has been implicated in attenuating AD progression. The findings of an exploratory study indicate that, in the plasma of AD patients, TREM2 expression is reduced in individual nuclear cells, whereas sTREM2 concentration is significantly increased. Elevated sTREM2 levels can affect TREM2 expression, thereby impacting the body’s ability to clear Aβ. sTREM2 concentration may therefore serve as a biomarker for early detection and evaluation of AD progression [180]. As mentioned earlier in the discussion, *APOE* and *TREM2* have been implicated in susceptibility to various viral infections (such as HIV and SARS-CoV-2). However, the current evidence remains insufficient for establishing a clear association between *APOE* or *TREM2* as predictive factors for AD and specific viral infections.

Although there is currently no definitive treatment for AD, there are ongoing developments in preventive strategies targeting viral infection. Based on epidemiological data, in countries with higher Bacillus Calmette-Guérin vaccination rates generally have a lower incidence of AD. This can be attributed to Bacillus Calmette-Guérin vaccine-associated increased systemic levels of IL-2, suppressed inflammatory responses, reduced Aβ plaque formation, and consequent restored AD-related cognitive function [181]. It has recently been suggested that GV971, a sodium oligomannate, suppresses gut dysbiosis and the associated phenylalanine/isoleucine accumulation. Furthermore, it can effectively reduce neuroinflammation and rescue the AD-related cognitive impairment [182]. Notably, sulfated polysaccharides exhibit an aptitude for binding with the S protein of SARS-CoV-2. This molecular liaison acts to curtail viral invasiveness, thus indicating a powerful antiviral activity [183]. Alternative forms of algal polysaccharides, particularly brown algal polysaccharides, have the ability to impede HSV-1-induced Aβ and tau formation when used synergistically with acyclovir. This finding expands the potential therapeutic scope of antiviral medications in the management of AD [184].

To date, mechanisms underlying the association of AD susceptibility genes with the pathogenesis of this brain disorder are still not adequately understood. While great efforts have been paid to the expression and function of such risk genes in histopathologic hallmarks of AD, explorations of AD susceptibility genes in inflammatory responses remain a highly contentious issue. Though it has long been recognized in both fundamental and clinical studies that abnormal inflammation exist in the pathologically vulnerable regions of AD brain, no anti-inflammatory agents have been translated into clinical applications. Considering the great complexity of inflammatory mechanisms occur in AD brains, the determination of any cause of inflammation may constitute an essential part of directions for future study. With the proposal of viral hypothesis of AD, investigations bridging studies of neuroscience and virology are of critical importance. Typically, how a specific virus interacts with AD risk genes, what kind of effects the virus-risk gene interaction may produce in the brain tissue, and how it would affect AD pathology, viral infection process and inflammatory reactions are all questions that require further evaluations. More importantly, the discovery and understanding of the genetic basis of AD may assist in the establishment of disease models for the development of treatment for both AD and infectious diseases.
